# Intercultural communication through the eyes of patients: experiences and preferences

**DOI:** 10.5116/ijme.591b.19f9

**Published:** 2017-05-16

**Authors:** Emma Paternotte, Sandra van Dulmen, Lindsay Bank, Conny Seeleman, Albert Scherpbier, Fedde Scheele

**Affiliations:** 1 Department of Healthcare Education, OLVG hospital, Amsterdam, the Netherlands; 2 NIVEL (Netherlands institute for health services research), Utrecht, the Netherlands; 3 Julius Center for Health Sciences and Primary Care, University Medical Center Utrecht, the Netherlands; 4 Institute for Medical Education, Faculty of Health, Medicine and Life Sciences, Maastricht University, the Netherlands

**Keywords:** Intercultural communication, patient-centred communication, patient perspectives, interviews, patient participation, the Netherlands

## Abstract

**Objectives:**

To explore patients’ preferences and experiences regarding intercultural communication which could influence the development of intercultural patient-centred communication training.

**Methods:**

This qualitative study is based on interviews with non-native patients. Thirty non-native patients were interviewed between September and December 2015 about their preferences and experiences regarding communication with a native Dutch doctor. Fourteen interviews were established with an interpreter. The semi-structured interviews took place in Amsterdam. They were focused on generic and intercultural communication skills of doctors. Relevant fragments were coded by two researchers and analysed by the research team by means of thematic network analysis. Informed consent and ethical approval was obtained beforehand.

**Results:**

All patients preferred a doctor with a professional patient-centred attitude regardless of the doctor’s background. Patients mentioned mainly generic communication aspects, such as listening, as important skills and seemed to be aware of their own responsibility in participating in a consultation. Being treated as a unique person and not as a disease was also frequently mentioned. Unfamiliarity with the Dutch healthcare system influenced the experienced communication negatively. However, a language barrier was considered the most important problem, which would become less pressing once a doctor-patient relation was established.

**Conclusions:**

Remarkably, patients in this study had no preference regarding the ethnic background of the doctor. Generic communication was experienced as important as specific intercultural communication, which underlines the marginal distinction between these two. A close link between intercultural communication and patient-centred communication was reflected in the expressed preference ‘to be treated as a person’.

## Introduction

Doctors in multicultural societies are increasingly confronted with patients from various ethnic backgrounds.^1^ The WHO emphasised the importance of a healthcare system that is capable to deliver healthcare from a patient-centred viewpoint for all kinds of patients.^2^ However, the cultural differences between doctors and patients challenge effective communication and the quality of care.^3^ Cultural influence on communication is well documented, mainly focussed on communication skills of doctors.^3^^-^^6^ There is limited literature focusing specifically on communication experiences and preferences of non-native patients.^7^ To improve the intercultural communication and subsequently the quality of care, insight into the communication process as experienced and preferred by non-native patients is needed.^8^^,^^9^

Literature shows that doctor-patient communication and patients’ perceptions of quality of care are influenced by the patient’s cultural views and language proficiency.^4^ Patients whose ethnic origins and cultural backgrounds are different from their doctor’s, evaluate the received care less positively than patients with the same background,^10^mainly because of communication problems resulting in lower mutual understanding and less satisfaction.^2^^,^^11^^,^^12^

A key-concept in research on doctor-patient communication is patient-centred care, a paradigm defined as care focused on the patient as a whole person with individual preferences situated within a social context.^13^ One of the key elements defining patient-centred doctor-patient communication is that doctors adapt their communication style to each patient’s preferences.^14^  The intercultural communication style of doctors could be seen as a combination of generic patient-centred communication skills and specific intercultural communication skills.^6^^,^^15^ Besides, a recent literature review of Degrie et al. mentioned the joined responsibility for intercultural communication of the patient and the caregiver, where non-verbal communication, the social dimension and cultural sensitivity of communication play a role.^9^ 

Despite extensive research on patient satisfaction,^8^ there is a lack of insight into patients’ preferences and experiences on intercultural communication.^7^^,^^9^^,^^12^^,^^16^ The latest review on minority patients’ experiences concluded that a broader perspective towards cultural sensitive care for all kinds of patients is desirable.^9^ Since shared decision making and patient-centred communication become more important in healthcare, patient’s preferences become more important as well.  Therefore, it is imperative to know more about non-native patients’ preferences regarding intercultural doctor-patient communication.^8^ Additionally, it is expected that better intercultural communication enhances patient involvement, satisfaction and health outcomes.^10^

The purpose of this study is to provide insight into patients’ preferences and experiences regarding their doctors’ communication in more detail. This could direct the development of intercultural communication training for doctors, which is not always structurally implemented in medical education.^3^^,^^17^ Therefore, we focused on two main research questions: Which kind of communication behaviours do non-native patients prefer in intercultural communication with their native doctors and how do they experience this communication? 

## Methods

### Study design

This qualitative semi-structured interview study was performed following the consolidated criteria for reporting qualitative research (COREQ criteria).^19^ Non-native patients were interviewed after visiting a native Dutch doctor in the Netherlands.

### Study participants

Non-native patients who visited a native Dutch medical specialist were asked to participate. Non-native patients were defined as ‘patients who were not born in the Netherlands or patients with at least one parent born outside the Netherlands’. If the patient did not speak Dutch, the interview questions and answers were translated by an interpreter. This interpreter could be a family member, another healthcare worker or a professional interpreter. If the patient was accompanied by family or other people, they were also involved in the interview.

All participants were informed about the aim and the procedure of the study beforehand. All participants signed informed consent. The study was performed in line with Dutch privacy legislation. Approval of the Dutch medical-education ethics board was obtained (NVMO-ERB 557). We confirm that all patient identifiers have been removed or anonymised so the patients described cannot be identified through the details of the story.

### Sample size

Of a total of 57 invited participants, 30 agreed to participate in the study. The most frequently mentioned reason to decline participation was lack of time. The interviews lasted between 5 and 30 minutes, depending on the participant’s available time and on the level of elaboration that could be achieved in the interview. Seven patients were available for a short interview, and in seven other interviews attempts to reflect on the question in a deeper way were unsuccessful, resulting in interviews that were shorter than 10 minutes. In interviews where reflection about preferences regarding the intercultural communication in general was unsuccessful, patients were asked to focus on the experiences of the last conversation with a Dutch doctor.

In total, 14 participants were accompanied by an informal interpreter. The other 16 participants did not need an interpreter. The ethnic backgrounds of the participants were Surinamese, Turkish, Moroccan, Portuguese, Indonesian, Iraqi, Irish, American and Chinese.

### Sampling procedure

The interviews, conducted in Dutch, were held between September 2015 and December 2015. Patients who met the inclusion criteria were asked to participate when they arrived at the outpatient clinic. To provide a heterogenic sample of medical specialties, the patients were selected at the outpatient clinics of 4 departments: gynaecology, internal medicine, urology and orthopaedic surgery. Patients were approached in the waiting room by the interviewer and were given sufficient time to decide before signing the informed consent form. After they had consulted the medical specialist, an interview took place in a separate room.

### Setting

Semi-structured interviews were conducted in a teaching hospital in Amsterdam, the Netherlands. This hospital was accounted as ‘migrant friendly’^18^ and around 70% of the patients in this hospital were non-native, as defined in section 2.2. Therefore, the doctors in this hospital were used to communicating in an intercultural context.

### Data collection procedure

The interviews were semi-structured and contained at least the following themes: preferences regarding the doctor’s behaviour, preferences regarding the doctor’s ethnic background, experiences regarding the influence of language and cultural differences on communication, general experiences regarding communication with doctors and, if this was difficult, their specific experience of the last consultation. The interviews were audiotaped and transcribed verbatim. After transcription, the audiotape was erased and the transcripts were anonymised.

### Data analysis

The transcripts were coded by attaching keywords (‘codes’) to all text fragments that were considered relevant to one of the research questions. To allow new insights, the coding of the interview transcripts was open and without a previously conceived coding schedule, using the program MAX-QDA. The codes were structured by means of thematic network analysis.^18^

Of the 30 transcripts, 9 were analysed independently by two members of the research team. To check reliability, differences in coding and selection of fragments were discussed in an iterative process until consensus about the content of the codes was reached. In this case, consensus was reached after discussing 5 transcripts. After coding 11 transcripts no new codes were derived. The developed coding scheme was discussed in depth among all authors. Results are structured by identified themes. Per theme, first patients’ preferences are presented, followed by their experiences. In the analysis, we focused on intercultural communication in general and did not differentiate per ethnic group.

## Results

### The characteristics of the doctor

All participants claimed that a doctor’s ethnic background was not important as long as the doctor was a professional. Some of the patients preferred a Dutch doctor instead of a doctor of their country of origin. The main reason for this claim was that many of the patients already lived in the Netherlands for a long time. The respondents described that they felt more Dutch than the ethnicity of their country of origin. Many patients mentioned that they experienced a difference between the healthcare system in the Netherlands and their country of origin.

“He needs to be a professional. Then I don’t have a preference regarding his background”. (Female, obstetrics department, interview 6)

Some participants had a clear preference for a doctor of a particular gender. Male as well as female participants said they had experienced feelings of shame when the doctor was of the opposite gender.

“As a male patient, I sometimes feel ashamed in front of a female doctor”. (Male, internal medicine department, interview 21)

On the other hand, other participants mentioned that if the doctor was a professional, the doctor’s gender was not an issue. Some patients expressed preferences for the age of a doctor. Some participants preferred older doctors, as they considered them to be more trustworthy.

### The doctor’s communication behaviour

Many participants mentioned that they felt comfortable when the doctor talked in an accessible way, such as: speaking slowly, using short sentences, explaining topics in various ways and avoiding medical jargon. Furthermore, participants considered it important that a doctor explains the diagnosis clearly, listens to patients, takes sufficient time, comforts the patient, gives advice and information to the patient and prepares the consultation beforehand. Furthermore, participants preferred an open and friendly doctor, who focusses his attention on the patient and not on the computer. Participants regarded a doctor who is honest about the diagnosis, as an example of open behaviour. An unfriendly doctor was described as someone who does not shake hands when greeting and who has a cold non-verbal attitude, such as leaning back in the chair. Also, doctors were experienced as friendly when, for example, the doctor asks patients to take a seat, before the real consultation starts.

“A friendly smile or something really simple can help to create a good atmosphere between the patient and the doctor”. (Female, obstetric department, interview 6)

Participants said that being treated as a unique person and not as a disease contributed to feeling satisfied with the medical consultation. They believed that communication was facilitated by acknowledgements, such as the feeling that the doctor understands the problem, and by a feeling of being important to the doctor. Patients expressed that when doctors asked more questions they felt respected and understood.

“Doctors need to create a connection with their patients, the doctor needs to trust the patient, which causes the patient to have a more open attitude”. (Male, orthopaedic surgery department, interview 30)

### Professional attitude and knowledge  

Participants repeatedly mentioned a doctor’s medical expertise, having enough time, and taking the problem of the patient seriously to be important. This was linked to the doctor’s professional behaviour, indicating that participants found their doctor to be a professional if he or she was medically up-to-date and well informed about possible treatment options.

Why should a doctor need to consult a book? A doctor should know such things, otherwise I can search for my own diagnosis in Google. (Female, obstetric department, interview 6)

It was frequently reported that doctors sometimes ask about their patient’s cultural habits and background. Many of the participants claimed to have no problem with this, especially when it was necessary for the doctor to know more about the background of the patient to be able to help them. However, a few participants mentioned feelings of discomfort in those situations because they were afraid the doctor would make assumptions about them.

### The doctor-patient relation

All participants mentioned that language differences were a challenge. Some participants said that communication problems were solved by the presence of an interpreter. Many patients preferred an informal interpreter. Many patients mentioned that it was the responsibility of the patient to speak Dutch more fluently.

“For me, a doctor is a doctor. The problem is the language”. (Male, internal medicine department, interview 24)

In intercultural communication, a good doctor-patient relation was mentioned by the participants as a facilitator for satisfactory communication. A good doctor-patient relation was for example established when the doctor and the patient knew each other for a longer period. Some participants said that many language differences seemed to have been solved when the doctor-patient relation was established. This was based on the experience that communication was easier if the participant and the doctor knew each other, because fewer words were needed to understand each other than during the first visit.      

All participants experienced positive feelings about the intercultural communication with their doctors and found it hard to come up with points of improvement for the doctor’s style of communication.

“I have never had a really unpleasant conversation with a doctor”. (Male, urology department, interview 11)

### Patient characteristics and participation skills

Some participants spontaneously reported that patient-doctor communication was also influenced by their own behaviour. Some participants were aware that their expectations may not always be clear for doctors, which could result in miscommunication. Also, participants considered it the patient’s responsibility to ask questions if they did not understand the doctor’s information about a diagnosis or treatment option. Participants stated that the communication could be influenced by patient characteristics, such as their educational level, religious beliefs and age.

### Knowledge of the healthcare organisation

The participants talked about the clarity of healthcare organisational aspects in the Netherlands. For example, some participants said they had initially been unaware that they needed a letter of referral from the general practitioner to see a medical specialist in the hospital. Also, a few participants were unfamiliar with the irregular availability of their doctor or the concept of a teaching hospital employing residents.

“I did not just have one gynaecologist or midwife. Instead, there was a different doctor every time”. (Female, obstetric department, interview 13)

## Discussion

The aim of this interview study was to explore non-native patients’ preferences and experiences regarding the intercultural communication with their native doctor. We found that the doctor’s ethnic background was considered as not important for this sample of non-native patients. However, a professional attitude of the doctor was very important for the patients. Furthermore, the patients preferred the doctor to focus on them as unique persons rather than only on the disease. Overall, the patients had positive experiences about the communication with their Dutch doctor, though a language barrier was mentioned as a major problem in an intercultural conversation. The patients stated that being acquainted with the doctor made language problems less prominent.

Some results of this study are well-known in literature, such as the language barrier as a problem in intercultural communication and the importance of professional attitude. However, a remarkable result of this study was that patients had no preference regarding the doctor’s ethnic background. We had expected that a doctor’s ethnic background would be important to patients. Concerning the effect of concordance in ethnic or racial background between the doctor and the patient, various effects have been found in the literature. On the one hand, it is concluded that race concordance was not important for the communication,^23^ which is confirmed by the patients in this study. While on the other hand, positive effects have been found of race or ethnic concordance between the doctor and the patient such as understanding the feelings of the patients when the doctor is of the same ethnicity.^24^ The fact that this was not the case in this study could serve as an argument against the proclaimed need for categorical care, where for example Turkish doctors care for Turkish patients.^25^ Many studies report about the positive effects of language concordance between the doctor and the patient.^21^^,^^22^ Since patients in our study mentioned language as the biggest barrier in a conversation with the doctor, we could imagine the positive effects of language concordance. However, the patients did not explicitly mention this.

In our study the importance of generic communication skills was showed. This is in line with the results of Mazzi et al. on the preferences of native patients, who identified relevant communication skills for doctors, such as listening attentively, treating the patient as a person and granting enough time.^8^ Although they did not investigate patient-doctor communication in an intercultural context, the similarity of the relevant communication skills could confirm that patient-centred communication is important in every context. In particular, the preference that ‘patients should be treated as a person’ was mentioned several times in our study. This is closely linked to the theory of patient-centred communication, which stipulates that every patient should be approached as a whole person.^13^^,^^26^ These results are also closely linked to the views expressed by the participants in our study. Considering that patient-centred communication seems to be relevant in an intercultural context, the relation between these two concepts of communication is of interest.^26^ The question whether patient-centred communication alone is sufficient enough for successful intercultural communication should be investigated in more depth.^26^^,^^28^ 

Patient-centred communication is not only an approach to guide doctors, it also asks something of patients’ participation, such explaining the reason of encounter.^28^^,^^29^ In our study the non-native patients seemed to be aware of this by mentioning the need of their own participation in a conversation. In addition, the patients mentioned unfamiliarity with the healthcare system as an issue.^26^ So, in intercultural communication it is important to account for the unfamiliarity of non-native patients regarding the healthcare system, which needs explicit attention in intercultural communication.^26^ 

During the interviews, the non-native patients in our study seemed to have difficulties in reflecting on their doctor’s communication behaviour. For example, participants mentioned that communication of doctors was most of the time good and they could sparsely formulate points of improvement. Based on this, we interpreted that these participants found it difficult to mention their preferences regarding the communication style of the doctor. Reflections on previous communication experiences were used to reflect at a deeper level. Still, the participants expressed mainly positive experiences. It could be, of course, that their doctors are already skilled intercultural communicators, since they all work in a ‘migrant friendly’ hospital,^18^ although there is always room for improvement. Other studies showed that patients were mainly positive about the communication with their doctors.^30^ The question remains whether patients, and especially non-native patients, have the capacity to reflect on their preferences or experiences regarding communication with their doctors at a deeper level and to formulate improvements. As a consequence, the results of the analysed data might be superficial. At the same time, insurmountable problems regarding intercultural communication probably would have been identified during the interviews, whereas more subtle intercultural communication issues need more profound reflection to be identified. Gaining more understanding on this issue is particularly important since patients are seen as important stakeholders in the evaluation of healthcare communication and patient’s views could guide training for doctors.^31^^,^^32^

The strengths of this interview study lie in the fact that we interviewed non-native patients, since patients are the ones who need to be satisfied with the doctor’s communication in order to experience good healthcare. Despite the effort to include non-native patients and to create a deeper level of interviewing with the non-native patients, the sample size was probably not big enough to create various deeper insights. Additionally, the various professional backgrounds of the researchers made it possible to reflect on the data from multiple perspectives. However, the interviews were performed by a Dutch interviewer, which may have influenced the responses. Further research should focus on the effect of the interviewer’s cultural background, in order to find out if a deeper level of understanding could be reached more easily between a patient and an interviewer who share the same cultural background. Another option to facilitate reflection is the use of films or observation of conversations.

The results in this study show an overlap of patient-centred communication and intercultural communication. Therefore, further research could focus on the distinction between these two and their overlap, which could facilitate further development of intercultural communication education for medical curricula.

To approach and learn every aspect of each culture that could inﬂuence the medical encounter is impractical, if not impossible, and reinforce stereotyping.^3^^,^^28^^,^^31^^,^^33^ We, therefore, chose to focus on the non-native patients as a group, instead of analysing the results according to their ethnic cultural background. However, a limitation is that the interviews were performed in one hospital in one country.

## Conclusions

Overall, non-native patients reported positive experiences regarding the communication with native Dutch doctors, and they did not prefer a doctor of a specific ethnic background. According to them, a language barrier constituted the most important problem, which would become less pressing once a doctor-patient relation is established. Generic communication of doctors was considered more important than specific intercultural communication, which could indicate the marginal distinction between intercultural communication and patient-centred communication. An additional conclusion is that reflecting on the communication skills of the doctor is difficult for patients.

### Acknowledgements

We would like to thank the participants for their time and willingness to share their preferences and experiences with the interviewer. We would like to thank Lisette van Hulst for her writing assistance and for editing the manuscript.

### Conflict of Interest

The authors declare that they have no conflict of interest.
